# Microfluidics Chip for Directional Solvent Extraction Desalination of Seawater

**DOI:** 10.1038/s41598-019-49071-7

**Published:** 2019-08-29

**Authors:** Hayder A. Abdulbari, Esmail A. M. Basheer

**Affiliations:** 1 Centre of Excellence for Advanced Research in Fluid Flow (CARIFF), Universiti Malaysia Pahang, Lebuhraya Tun Razak, 26300 Gambang, Kuantan, Pahang Malaysia; 2 Faculty of Chemical & Natural Resources Engineering, Universiti Malaysia Pahang, Lebuhraya Tun Razak, 26300 Gambang, Kuantan, Pahang Malaysia

**Keywords:** Chemical engineering, Surface patterning

## Abstract

Directional solvent extraction is one of the promising membrane-less seawater desalination method. This technique was not extensively investigated due the poor mixing and separation performances of its bench-scale system. It is believed that, overcoming these drawbacks is possible now with the rapid development of microfluidics technology that enabled high-precession micro mixing and separation. This work presents microfluidics chip for extracting and separating salt from seawater. The chip was designed with two sections for extraction and separation. In both sections, the liquids were separated using capillary channels perpendicular to the main stream. The main channels were designed to be 400 µm in width and 100 µm in height. Two streams inlets were introduced through a Y-junction containing octanoic acid as the organic phase and saltwater as the aqueous phase. The desalination performance was investigated at four different temperatures and five different solvent flow rates. Water product salinity was recorded to be as low as 0.056% (w/w) at 60 °C and 40 mL/h. A maximum water yield of 5.2% was achieved at 65 °C and 40 mL/h with a very low solvent residual (70 ppm). The chip mass transfer efficiency was recorded to be as high as 68% under similar conditions. The fabricated microfluidic desalination system showed a significant improvement in terms of water yield and separation efficiency over the conventional macroscale. The high performance of this microsystem resulted from its ability to achieve a high mixing efficiency and separate phases selectively and that will provide a good platform in the near future to develop small desalination kits for personal use.

## Introduction

Directional solvent extraction desalination (DSE) was first introduced by Davison and coworkers in 1960s as an alternative, membrane-free desalination technique, where water is dissolved in a solvent at high temperature and recovered by decanting it at low temperature^1^. Theoretically, there are few organic solvents with high polarity and insolubility in water that are capable of dissolving the water while discarding other impurities^2,3^. Hence, a high-polarity functional group, such as carboxylic acid, is best used to contemplate this characteristic, specifically the Carboxylic edible oils. The hydrophilicity of carbon with double-bonded oxygen and single-bonded hydroxyl chains allows H-bonds to be formed with water molecules. Whereas, the hydrophobicity of the fatty acid in the chain promotes salt rejection. Despite all the promising advantages of this technique, it is not further investigated or commercially applied due to two major drawbacks that affect its efficiency. The first drawback is the massive and complicated mixing and separation steps^4,5^. A study conducted by Sanap *et al*. showed that the contactor, a utility, used to extract water into the solvent was under severe time pressures and permitted only three stages of liquid contact, which led to greater energy needs^6^. Additionally, the separation is simply a settling process, thus requires time and huge space. The second drawback is the existence of solvent residuals in the effluent product water due to the poor selective decanting technique which relies on gravitational forces^7^.

Microfluidics is a multidisciplinary technology which integrates several fields, including chemistry, biology, biotechnology, and medicine^8–10^. Microfluidics devices have shown a rapid development owing to its large surface area-to-volume ratio and is therefore advantageous for many applications^11–17^. One of the important features provided by microfluidics is the micro-mixing of fluids at the smallest scales^18–20^. Microfluidics’ micro-mixing techniques, devices, and efficiency have been investigated extensively by many researchers in attempts to optimize performance and explore possible applications further^21–24^. Having defined, in the case of the fluids flowing in microchannels, mixing is driven by molecular diffusion proceeding in deformed fluid elements. Therefore, for a virtually perfect mixing to occur, expanding the space which allow the diffusions between the two species seems to be a vital option for micro size mixing. This has been one of the advantages of having the process in micro size which clearly impossible to be achieved in macro size mixing. In microfluidic mixing devices, fluids can typically be mixed within 55–300 ms and hence the requirement for high device throughputs can be readily achieved with a very simple structure^22^. For that characteristic, microfluidics is believed to be an alternative approach for other application such as macroscale directional solvent extraction. Besides, microfluidics devices have shown a remarkable performance in specific phase separation^25,26^. At the macroscale, the phase separation is driven by the density difference between the phases and thus by the difference in gravitational forces. However, gravitational forces are negligible in microfluidics, which makes a complete separation of two phases in a single step using surface forces possible^18^. In a study conducted by Castell and coworkers, microfluidics system for separating two immiscible liquids (chloroform and water) yielded an outstanding performance with 100% separation efficiency. According to their findings, the capillary section used to separate the liquids utilized the differences in the two phases wettability due to the advantage of miniaturized systems where the surface and intermolecular forces dominated.

Capillary action is one of the factors affecting the flow of fluids in microchannels and is governed by adhesive and cohesive forces. Mainly, adhesive forces involve the contact between the fluids and tube’s surfaces whereas cohesive forces comprehend the interactions of the fluids within the tubes. Both forces are directly related to the surface tension in such a manner that when there are higher adhesive and lower cohesive forces, the surface tension is decreased, leading to better capillary action. In addition, the size of the tubes and gravitational forces are also crucial in the capillary effect. Capillary microfluidic devices use capillary effects to manipulate liquids. Capillary microfluidics is often considered to be ‘passive’ because it typically employs no real-time control over the flow, whereas ‘active’ devices exert external peripheral control^11^. There are numerous different methods for using capillary effects for flow control in microchannel-based microfluidics^13,27–29^. There are a number of reported works in the field of microfluidics that have used capillary effects in biological applications. Zimmermann and colleagues used 15 tree-like capillaries to pre-programmed immunoassays^29^. Similar automated liquid handling systems were used by other researchers later on for bioassays. The applications of capillaries in such a dynamic and specific routine is a key advancement in the microfluidics developments. Thus, it is possible to utilize it in other applications such as separations.

It is believed that, the unique mixing and separation features provided by microfluidics technology will enable revisiting the DSE method and addressing the two major drawbacks of its bench scale system. The present work introduces the first microfluidic system for directional solvent extraction desalination of saltwater with capillary separations. A microfluidics chip was designed to have two sections. The first section is where extraction takes place and the second section is where the solvent is recovered. The design used a separation capillary at the end of each section. These capillaries selectively separated the two phases based on their hydrophobicity–hydrophilicity nature.

## Results and Discussion

**Figure 1:**
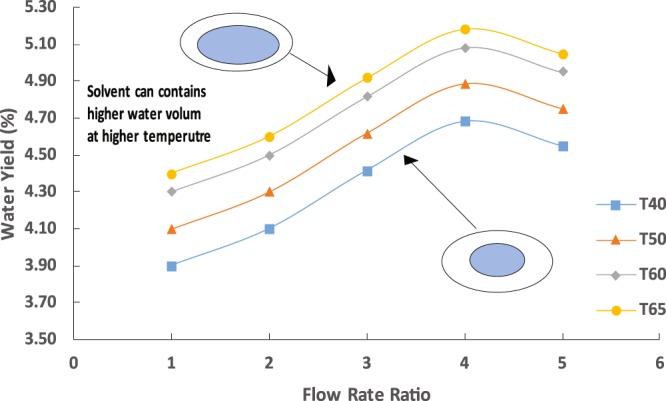
Water yield at different temperatures using the capillary chip for fixed aqueous solution flow rate of 10 mL/h.

The chip performance in extracting water from saltwater was evaluated in four important aspects: product yield, salinity, solvent residuals, and mass transfer efficiency. The changes in the product yield as a function of the system operating temperature and total flow rate are presented in Fig. 1, where the water yield linearly increased with the increasing temperature and the maximum water yield was recorded (5.2%) at the highest temperature. This is consistent with the mass transfer theory where in a liquid–liquid extraction, the solute moves from inside the aqueous phase to the surface through diffusion or capillary action which is caused by the differences in the wettability of the two phases, and once at the surface, it is limited by the convective mass transfer. High temperature leads to high mass transfer, facilitating the extraction process and increasing the extraction yields^30^. Fundamentally, molecular diffusion is caused by thermal random motion in the solution. This motion, however, is affected directly by the changes in the temperature of the solution. A few other factors can contribute to changes in this motion as well, such as pressure; molecular properties, i.e., mass and volume; and the forces between molecules. Such an effect was observed clearly when the temperature of the solution increased; there was a significant increase in the water yield as a result of the increase in diffusion. The effect can also be explained as a result of the reduction in the viscosity and surface tension of the solvents at a higher temperature, thus enabling the solute to reach active sites inside the solvent matrix easily and extract more water.

In contrast, the water yield increased with the increasing total flow rate which was represented by the flow rate ratio and reached maximum at 40 mL/h. Five different flow rates for the solvent were examined (10, 20, 30, 40, and 50 mL/h), while the saltwater flow rate was fixed at 10 mL/h. The ratios of solvent-to-saltwater flow rates are denoted as the flow rates 1, 2, 3, 4, and 5, respectively. A further increase in the total flow rate beyond 40 mL/h (represented by the ratio of 4) reduced the water yield due to the short mixing time for the solvent with the saltwater for sufficient mass transfer. For a long channel with diameter d (µm), the time required for mixing due to molecular diffusion in a laminar flow across the channel can be estimated by the following equation^30^:

1 $${t}_{mix}=\frac{d{h}^{2}}{D}$$

where dh is the hydraulic diameter (m2) and D is the diffusion coefficient (4 × 10^−9^ m2/s). At 50 mL/h, the required mixing time of the fluids calculated using Eq. 1 was 6.4 s, whereas the residence time of the fluids in the channel was 7.8 s, obtained from the residence time distribution (RTD) study at the given flow rate. As shown in Fig. 1, the maximum water yield obtained was 5–5.20% at the extraction temperature of as low as 65 °C and total flow rate of 40 mL/h. As reported in earlier studies^3,31^, water desalination using octanoic acid as directional extraction solvent resulted in a water yield of as high as 3.8% only, which is lower than that obtained in the current microfluidics system. The low performance of the system was due to the strong interactions of water with the dissolved salt and the resulting difficulty of extracting water from a salt–water solution. Fundamentally speaking, the larger the water droplet in the octanoic acid, the slower the diffusion^32^. However, in macrosize, the droplets are often large and uncontrollable, thus longer time is needed for adequate diffusion. In contrast, microfluidics has the advantages of controlling the droplet size at a very small size.

**Figure 2:**
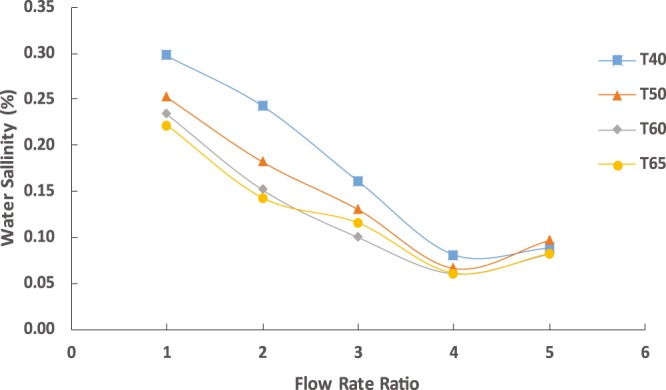
Water salinity at different temperatures using the capillary chip for fixed aqueous solution flow rate of 10 mL/h.

The salinity of the product water was recorded and plotted against the changes in the extraction temperature and total inlet flow rates. As shown in Fig. 2, water salinity decreased dramatically with the increase in the total flow rate up to 40 mL/h. A further increase resulted in an increase in the water product salinity. As discussed earlier, the high flow rate, i.e., 50 mL/h, reduced the residual time for the liquids, thus less contact time between the fluids. The salinity also decreased with the increasing temperature. For example, at 40 °C and 10 mL/h, the salinity was 0.3% and reduced to 0.27% at 65 °C and the same flow rate. However, the factor in decreasing the salinity of the product is how efficient is the separation of the system. In other words, the salt contained in the product water is mainly controlled by the separation process as explained by Bajpayee^7^. In contrast, the water salinity significantly decreased at low temperatures and flow rates. For instant, at 40 °C and 10 mL/h, the water salinity was 0.31% and reduced to 0.24% (22% improvement) when the temperature was increased to 50 °C at the same flow rate. On the other hand, the water salinity was 0.07% at 50 °C and 40 mL/h and decreased to 0.06% at 60 °C at the same flow rate (14% improvement). This can be attributed mainly to the flow rate changes where a limited contact time can be achieved between the solvent and saltwater at high flow rates. The reported salinity of the product water using the macrosize system was as high as 0.20% at similar conditions, which is four times higher than that obtained in our microfluidics system. This low salinity of the microfluidics chip is much higher than that obtained in the large scales. As reported in earlier studies, the salinity of the water product obtained at the macroscale was around 0.23% (76% improvement using the microchip)^7^.

**Figure 3:**
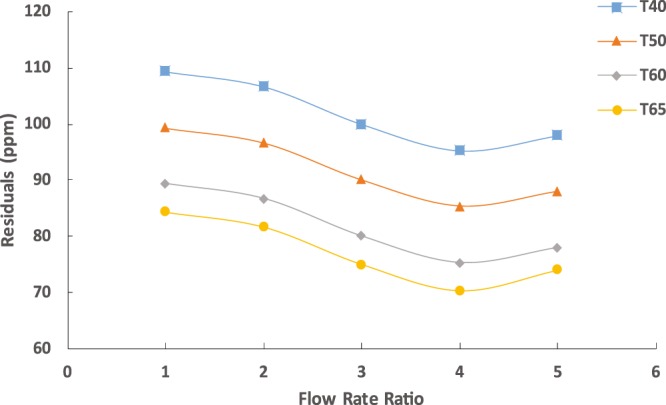
Solvent residuals in product water at different temperatures using the capillary chip for fixed aqueous solution flow rate of 10 mL/h.

Figure 3 shows the amount of solvent residuals in the water product at different temperatures and inlet flow rates. The solvent residuals decreased linearly with the increasing temperature possibly due to the increase amount of water diffused into the solvent at the same rate of solvent dissolving in the water. Thus, low residuals concentration was obtained at high temperatures. On the other hand, the residuals decreased with the increasing flow rate in the range of 10–40 mL/h and declined beyond that due to the low water yield at these conditions.

**Figure 4:**
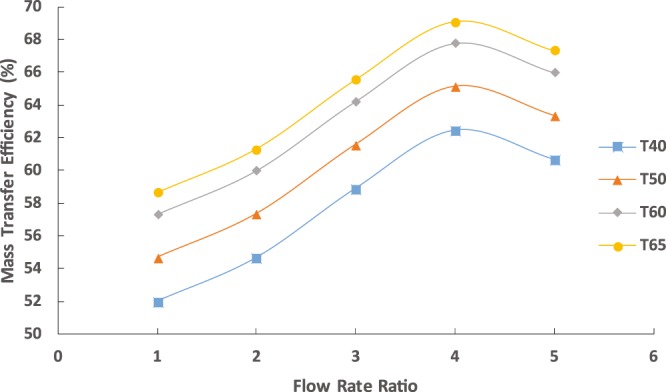
Mass transfer efficiency of the chip at different temperatures using the capillary chip for fixed aqueous solution flow rate of 10 mL/h.

Figure 4 shows the mass transfer efficiency of the fabricated system at different temperatures and inlet flow rates. The mass transfer efficiency of the system linearly increased with the increasing flow rate ratio within the range of 1–4. A further increase reduced the efficiency due to the short contact time between the two phases which resulted in a poor mass transfer. The mass transfer efficiency increased dramatically with the increasing temperature. The increase in temperature leads to an increase in the solute diffusion, which results in increasing the mass transfer.

**Figure 5:**
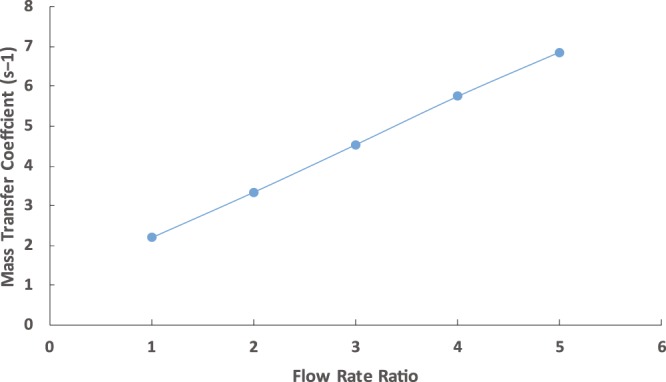
Mass transfer coefficient using the capillary chip for fixed aqueous solution flow rate of 10 mL/h.

An overview of a similar works on mass transfer within the segmented flow are listed in Table 1. There is an inconsistency in the used definition for k_La_. As shown in Table 1, the fabricated chip showed a high overall volumetric mass transfer due to the length of the channel which was long enough for a good contact between the two phases. As shown in Fig. 5, the mass transfer coefficient increased with the increasing flow rate ratio due to the decrease in the droplet size which enhanced the mass transfer.

**Table 1:** Mass transfer within segmented flow.

Reference	dh*	Max. kLa
Ghaini^39^	1000	1.30
Kashid *et al*.^33^	400	0.30
Dessimoz *et al*.^34^	400	0.50
Assmann and Rohr^40^	220	2.70
Raimondi *et al*.^41^	210	0.30
Kralj *et al*.^42^	157	5.30
Current work	160	7.03

*dh is the hydraulic diameter of the microchannel.

**Figure 6:**
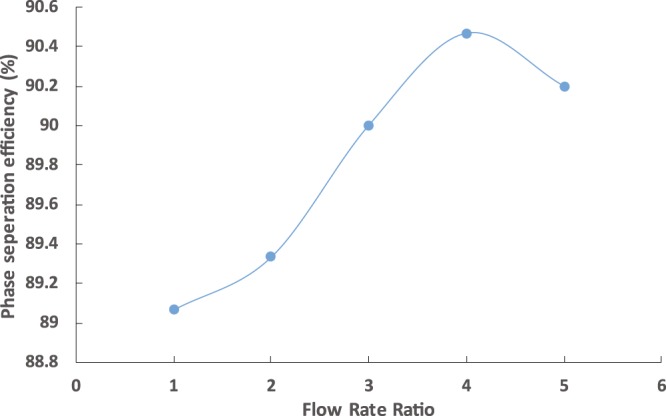
Phase separation efficiencies of the chip at different temperatures using the capillary chip for fixed aqueous solution flow rate of 10 mL/h.

An important induction of the chip performance is the phase separation efficiency, which can be obtained by calculating the initial weight of the solvent and the recovered as described by Eq. 2. The separation efficiency of the capillary in separating the two phases at different flow rate ratio is presented in Fig. 6. The separation efficiency was 89% at a flow rate ratio of 1 and increased slightly to 90% at a flow rate ratio of 4. A further increase in the flow ratio did not change the separation efficiency because the number of capillaries was not sufficient to further separate the phases. However, the separation efficiency obtained in this study was in the range of 89%–90%, which was high enough to reduce the salt and solvent residuals in the water product. The solvent residuals had concentrations as low as 70 ppm, and the salinity was 0.056%. In comparison, the earlier macroscale experiments reported solvent residual concentrations and salinity of 700 ppm and 0.23%, respectively, indicating that miniaturizing the processes has drawbacks.

2 $$Phase\,Sep.\,Eff=\frac{{m}_{i}-{m}_{r}}{{m}_{i}}\times 100$$

Where ${m}_{i}\,$is the initial weight of feed solvent and ${m}_{r}$ is the weight of the recovered solvent.

## Conclusions

Directional solvent extraction desalination method was briefly investigated in the past few decades as one of the promising membrane-less desalination techniques due to two major drawbacks (poor mixing and separation performances). The unique characteristic of micro-flow systems that are controlled by the transported liquids physical properties provided good opportunity to re-investigate the directional solvent extraction desalination method with more efficient mixing and separation environment. A directional solvent extraction desalination of seawater in a capillary microfluidic chip is presented in this paper. The chip was designed with two connected sections for micro mixing and micro separation of the two partially miscible phases (fatty acid and saline water). The mixing of the two phases was achieved by introducing a high-precession droplets generation section that allows controllable dispersion of the saline water droplets in the octanoic acid media and that provided high mass transfer area for enhanced water extraction performance. The two phases solvation was controlled by controlling the operation temperatures in the mixing and separation sections for maximum desalination performance. To overcome the second drawback (separation performance), two capillary sections were used to efficiently separate the extract water, residual saltwater and the solvent. Using capillary action improved the water yield up to 47% at the average temperature and maximum solvent flow rate. Additionally, the product water’s salinity was reduced with via capillary separation to as low as 0.056%, which is 78% lower than the salinity of the water product obtained at the macroscale. In addition, the solvent residuals were reduced when using the microfluidic chip, and their concentrations were found to be as low as 50 ppm. Based on the results, it is believed that with an appropriate temperature and integrated automated system, this chip can be a future replacement for the current desalination methods.

## Methods

### Materials

Octanoic acid was used as the organic phase (solvent) due to its high ability to dissolve more water than decanoic acid^31^. Sodium chloride (99%, fine powder) was used to prepare the saltwater with a salinity of 3.5% (aqueous phase). Polydimethylsiloxane (PDMS) was used to fabricate the microfluidic chip. All chemicals were purchased from Sigma-Aldrich.

### Chip design and fabrication

**Figure 7:**
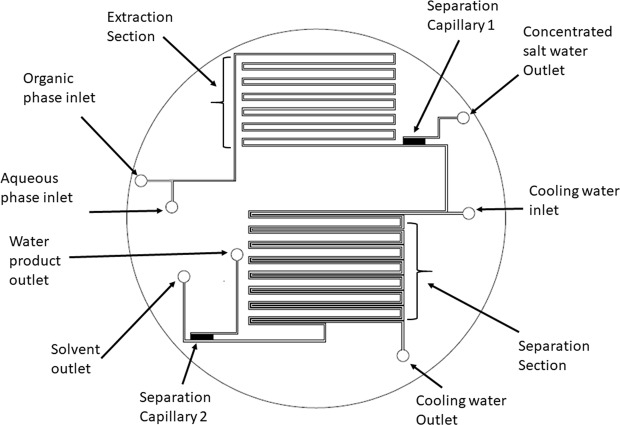
Complete Microchip Design.

**Figure 8:**
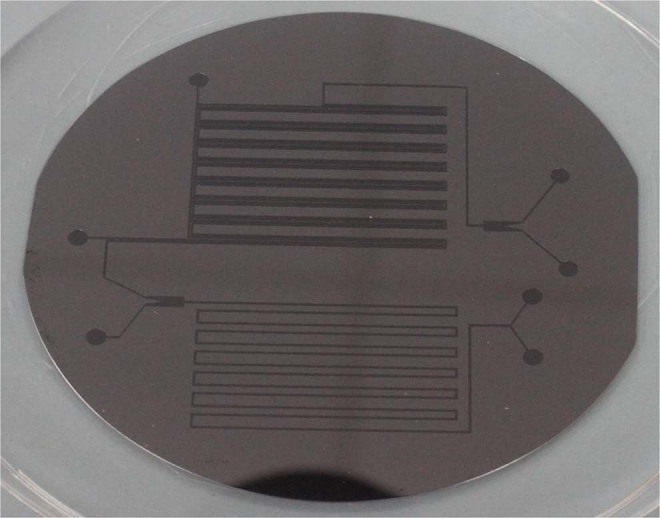
DSE capillary desalination chip.

**Figure 9:**
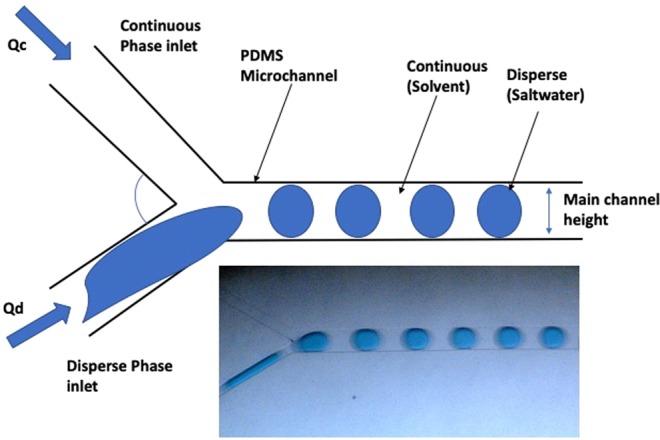
Droplets generation and flow after the T-junction.

**Figure 10:**
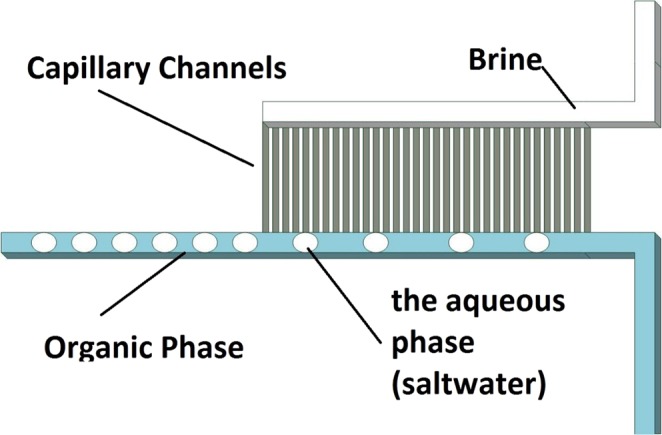
Illustration of capillary brine separation section.

Figures 7 and 8 show the design of the microfluidics chip. The chip was designed to have two major sections namely extraction and separation sections. The extraction section started by creating a Y-junction where the aqueous phase (saltwater) was injected into the organic phase (octanoic acid). The flows of the two phases were carefully controlled using pressure and vacuum controller pumps to ensure uniform droplets generation through the channel (Fig. 9). The droplets passed through the multiple turns extraction section that was designed for a droplet residence time of 32, 17, 12.6, 9.8, and 7.8 s for 10, 20, 30, 40, and 50 mL/h flow rates, respectively, and with 13 turns. The outlet of the extraction section is connected to a capillary separation section that was specially designed and fabricated to separate the brine from the solvent by utilizing the differences in wettability properties of both phases as shown in Fig. 10. This section consists of 100 capillaries (the capillaries length is 2000 μm and the width is 3 μm with a spacing of 50 μm), which are located to be perpendicular to the main channel (Fig. 5). The extracted water was decanted from the solvent by cooling down the mixture using a parallel channel (cooling channel, which has the same dimensions as the main channel of 400 μm in width and 100 μm in height). At the end of the section, a second separation capillary is used to separate the solvent and the product water, which has the same dimensions as the first capillary. It is important to highlight that all channels except the capillary are 400 μm width and 100 μm height, with a fixed depth of 100 μm. The PDMS chip fabrication procedure is similar to the one we implemented previously^20^.

**Figure 11:**
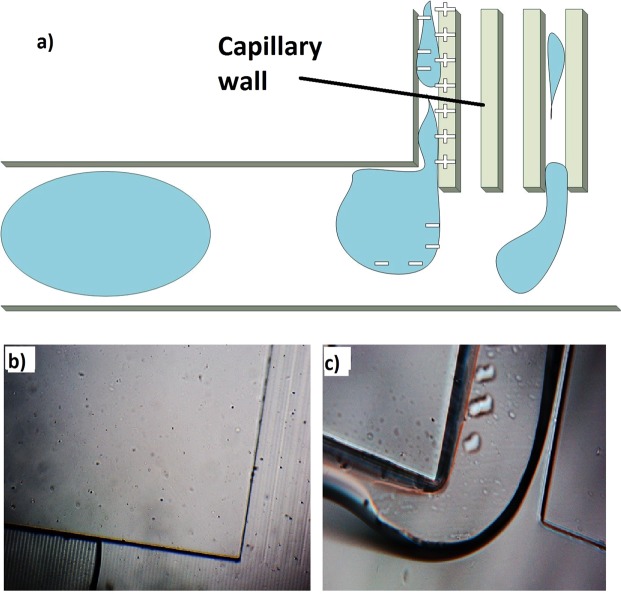
Wettability mechanism for brine separation. (**a**) Three-dimensional (3D) illustration of the mechanism, (**b**) microscopic image of the capillary section, and (**c**) microscopic image of one capillary and the flow through it.

**Figure 12:**
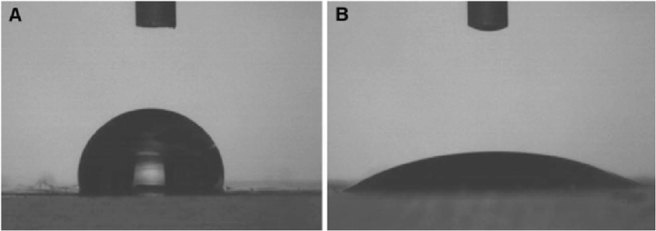
PDMS wettability by (**a**) water and (**b**) octanoic acid with a droplet volume of 10 µl.

A wettability difference between the two phases plays an important role in the operation of the device. As the two phases go through the separation section (capillary), separation can take place based on their wettability differences as shown in Fig. 11. Octanoic acid was observed to wet the channel walls, whereas water was repelled by the high hydrophobic PDMS surface. This behavior can be observed by the contact angle test of the two liquids on the surface of PDMS. As shown in Fig. 12a, water had a sharp angle on the PDMS surface as hydrophobic behavior. In contrast, octanoic acid was more hydrophilic as shown in Fig. 12b. This disparity between the two liquids is with maintaining low pressure at the capillary is adequate to separate them. The capillary outlet was connected to a syringe pump to maintain the pressure at 13 kPa.

### Capillary theory

In micro-flows, such as in microfluidic devices, capillary action is utilized to either separate or mix liquids. Both situations take advantage of the ability of liquids to rise vertically in very narrow sub-paths to heights which depend on their surface tensions and the geometry of the paths. The liquids in this study are transported through the microchannels by the mean of capillary pressure or capillary action.

As mentioned, the capillary pressure can be varied with the shape of the narrow paths. Most microfluidic devices are rectangular in shape since they are commonly made with planar photolithography or rapid prototyping methods which usually default to the rectangular shape. Therefore, the YL (Yong-Laplace) model can be used to estimate the capillary pressure of these tiny pathways using the relationship between the contact angle of the liquid/microchannel wall and microchannel size. This relation is as expressed in the following equation:

3 $$P=-\,\gamma [\frac{\cos \,{\theta }_{t}+\,\cos \,{\theta }_{b}}{h}+\frac{\cos \,{\theta }_{1}+\,\cos \,{\theta }_{r}}{w}],$$

where P is the capillary pressure, γ is the surface tension of the liquid in the microchannel, h is the channel height is the channel width, and θ_t_, θ_b_, θ_l_, and θ_r_ are the top, bottom, left, and right contact angles of the liquid with the corresponding four microchannel walls.

For the proper operation of the capillary channels (CC) during evaluation, it is important to consider some practical aspects. Firstly, the measured contact angle should be less than 90° to ensure high capillary pressure since the capillary pressure becomes insignificant at moderate to large contact angles (approaching 90°). Additionally, when the contact angle is large, the surface tension will be lower, thus impacting the resulting capillary driving force (Eq. 3). Therefore, it is recommended that the contact angle should be less than or equal to 60° to ensure that the designed capillaries will yield high functionality. Secondly, it is important to note whether the channel materials are homogenous or not. When the four walls of a vertical microchannel are made of the same material, the contact angle will be even all directions. On the other hand, for sandwich and sub-combined channels, the contact angle can vary based on the materials used. In fact, the majority of fabricated microfluidic devices are sealed with different materials and often have different contact angles. Thus, there are some difficulties associated with estimating the capillary pressure in such devices. Nevertheless, the YL model can still feasibly estimate the pressure or capillary behavior but with less accuracy.

### Mass transfer mathematical models

Mass transfer efficiency E in the device is quantified according to Eq. (4)^26^. This describes the concentration difference achieved between the channel inlet and outlet (numerator) compared to the maximum possible concentration difference defined by the equilibrium bulk concentration or the amount transferred over the maximum amount transferable. The equilibrium bulk concentration is derived from the partition coefficient K as in Eq. (5), which is defined as the ratio of equilibrium concentrations in the organic phase to the aqueous phase^33^.

4 $$E=\frac{{C}_{1}^{out}-{C}_{1}^{in}}{{C}_{1}^{eq}-{C}_{1}^{in}}$$

5 $$K=\frac{{C}_{1}^{eq}}{{C}_{2}^{eq}}$$

where C is the concentration (mol/L), the subscript 1 is for the continuous phase (organic), and the subscript 2 is for the dispersed phase (aqueous). The superscripts are for inlet, outlet, and equilibrium bulk.

Previous studies reported an important measurement for continuous mass transfer devices using the volumetric mass transfer coefficient (s^−1^), which is a product of mass transfer coefficient (kL) and specific interfacial area (a)^34–36^. The specific interfacial area is defined as the interfacial area per unit volume of the dispersed phase [m2 m^−3^]. Eq. 6 is used for k_La_^37^:

6 $${k}_{L}a=\frac{1}{\tau }\,\mathrm{ln}(\frac{{C}_{1}^{out}-{C}_{1}^{in}}{{C}_{1}^{eq}-{C}_{1}^{in}})$$

where τ is the residence time (s) (the residence time is calculated using RTD measurement) and C is the inlet and the outlet concentrations (g/mol).

### Experiment procedure

**Figure 13:**
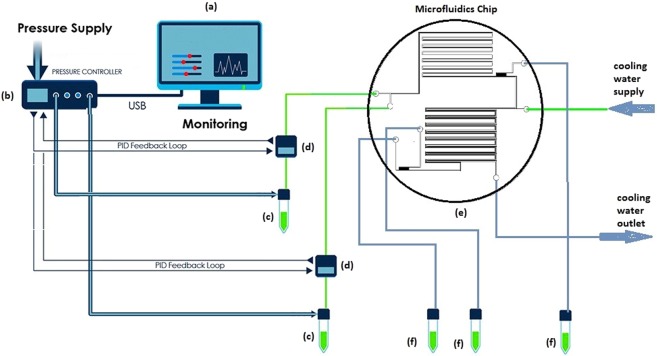
Schematic diagram of the experimental setup: (**a**) computer installed with Elveflow Smart Interface, (**b**) pressure and vacuum controllers connected to a compressor, (**c**) reservoirs containing the solution, (**d**) flow sensors, (**e**) custom-made microchannel, and (**f**) beakers as the collecting tanks.

Figure 13 shows the experimental setup for obtaining experimental data at all liquid ratios. Saltwater and solvent were pumped into the PDMS chip from two separated reservoir tanks 100 mL each connected to ElveFlow pressure and vacuum controller pump (model OB1 Mk3 200). All the setup equipment and the PDMS chip were connected using microfluidic PTEF Teflon tubes (Polytetrafluoroethylene, Teflon® tubing) with a diameter of 1/s inch. The outputs of the two reservoirs were directly connected to two flow meters (ElveFlow flow sensor) for the flow rate measurements. These flow meters were calibrated by the supplier (ElveFlow) with a maximum reading error of 0.15%. The two liquids were then directly connected to the PDMS chip in Tanks 1 and 2 (Fig. 7e) using microfluidic PTEF Teflon tube. There are three main outlets after flow namely, brine, product water, and solvent outlets. The first outlet, where the concentrated saltwater (brine) was collected, is located after the first capillary section. At the other two outlets, the product water and solvent were collected after the second capillary section.

In the present work, the temperature effect on the chip performance was evaluated at 40, 50, 60 and 65 °C (denoted as T40, T50, T60, and T65, respectively). This range of temperature was chosen to avoid any complication with the PDMS chip that is sensitive to high temperature. At each temperature, five different flow rates of solvent were examined (10, 20, 30, 40, and 50 mL/h), while the saltwater flow rate was fixed at 10 mL/h. The ratio of solvent-to-saltwater flow rate are denoted as 1, 2, 3, 4, and 5 flow ratios. The water salinity at the output of the microfluidic desalination chip was evaluated and monitored using a conductivity meter. Initial calibration was done prior to measurement. When evaluating the chip performance, the water product yield was calculated by monitoring the ratio of the mass weight of the recovered water (wp) to the mass weight of the octanoic acid (ws) described in Eq. 7.

7 $$Y=\frac{{W}_{p}}{{W}_{s}}$$

**Figure 14:**
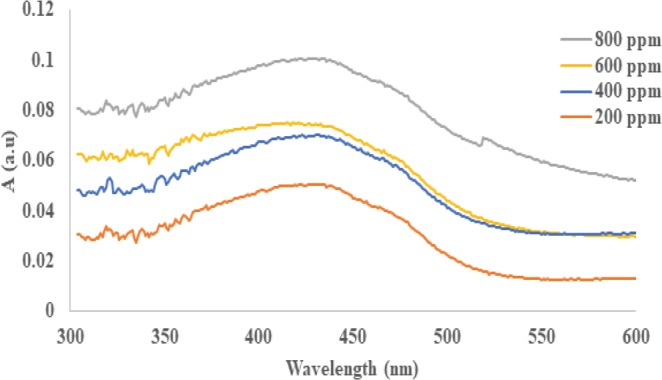
Calibration curves for the UV-Vis absorption peaks for four different concentrations of acid.

To evaluate the solvent losses, samples of the water product were analyzed using a UV-Vis spectrometer. The analysis was conducted to determine the amount of the solvent residue in the water product by observing the changes in the absorption peak value at a wave length of 434 nm. The calibration curve for this analysis was prepared prior to the experiment, as shown in Fig. 14 ^3,31,38^.
